# Rab6a/a’ Are Important Golgi Regulators of Pro-Inflammatory TNF Secretion in Macrophages

**DOI:** 10.1371/journal.pone.0057034

**Published:** 2013-02-21

**Authors:** Massimo Micaroni, Amanda C. Stanley, Tatiana Khromykh, Juliana Venturato, Colin X. F. Wong, Jet P. Lim, Brad J. Marsh, Brian Storrie, Paul A. Gleeson, Jennifer L. Stow

**Affiliations:** 1 Institute for Molecular Bioscience, The University of Queensland, Brisbane, Queensland, Australia; 2 Department of Biochemistry and Molecular Biology and Bio21 Molecular Science and Biotechnology Institute, The University of Melbourne, Victoria, Australia; 3 The Department of Physiology and Biophysics, University of Arkansas for Medical Sciences, Little Rock, Arkansas, United States of America; Institut Curie, France

## Abstract

Lipopolysaccharide (LPS)-activated macrophages secrete pro-inflammatory cytokines, including tumor necrosis factor (TNF) to elicit innate immune responses. Secretion of these cytokines is also a major contributing factor in chronic inflammatory disease. In previous studies we have begun to elucidate the pathways and molecules that mediate the intracellular trafficking and secretion of TNF. Rab6a and Rab6a' (collectively Rab6) are *trans*-Golgi-localized GTPases known for roles in maintaining Golgi structure and Golgi-associated trafficking. We found that induction of TNF secretion by LPS promoted the selective increase of Rab6 expression. Depletion of Rab6 (via siRNA and shRNA) resulted in reorganization of the Golgi ribbon into more compact structures that at the resolution of electron microcopy consisted of elongated Golgi stacks that likely arose from fusion of smaller Golgi elements. Concomitantly, the delivery of TNF to the cell surface and subsequent release into the media was reduced. Dominant negative mutants of Rab6 had similar effects in disrupting TNF secretion. In live cells, Rab6–GFP were localized on *trans*-Golgi network (TGN)-derived tubular carriers demarked by the golgin p230. Rab6 depletion and inactive mutants altered carrier egress and partially reduced p230 membrane association. Our results show that Rab6 acts on TNF trafficking at the level of TGN exit in tubular carriers and our findings suggest Rab6 may stabilize p230 on the tubules to facilitate TNF transport. Both Rab6 isoforms are needed in macrophages for Golgi stack organization and for the efficient post-Golgi transport of TNF. This work provides new insights into Rab6 function and into the role of the Golgi complex in cytokine secretion in inflammatory macrophages.

## Introduction

Macrophages secrete cytokines as part of innate immune responses [1]. Inflammatory cytokines, including the potent tumor necrosis factor (TNF), help to recruit and activate other cells to orchestrate protective action against infectious microbes or other stimuli. TNF and other inflammatory cytokines are also linked with the exacerbation of inflammatory disease [2] and in this guise they are important therapeutic targets for the clinical management of a widening range of diseases.

The secretory pathway and trafficking machinery used by macrophages to traffic newly made cytokines to the cell surface for release are gradually being elucidated. The *trans*-membrane precursor of TNF is trafficked through the Golgi where its exit from the *trans*-Golgi network (TGN) occurs in tubular carriers for transport to recycling endosomes and thence to filopodia and phagocytic cups at the cell surface for cleavage and release [3], [4]. Lipopolysaccharide (LPS) up-regulates the expression of specific membrane fusion proteins [5], [6], [7] and increases the budding of TGN-derived membrane carriers to facilitate TNF trafficking and secretion [8], [9]. Specific trafficking molecules of the SNARE and Rab families that mediate post-Golgi transport and TGN-associated golgins have been identified as regulators of TNF trafficking in macrophages [10], [11].

Members of the Rab family of small GTPases mediate the specificity between donor and acceptor membranes for vesicle budding, docking, tethering and fusion in transport steps throughout eukaryotic cells [12], [13], [14]. This model predicts that multiple Rab proteins will be sequentially engaged for the trafficking of cytokines in macrophages. The Golgi complex is a focal point for the trafficking of several secreted cytokines and here we investigate Rab6 as the quintessential and most abundant Golgi-associated Rab protein [15]. It consists of four different isoforms (Rab6a, a', b and c). Rab6a and Rab6a', which are equally abundant and differ in three amino acids, are ubiquitously expressed in cells [16], whereas Rab6b is restricted to neuronal tissue [17] and Rab6c is expressed in a limited number of human tissues and is involved in cell cycle progression [18]. Rab6 proteins regulate anterograde and retrograde traffic at the level of the Golgi complex via interactions with numerous and unrelated effector proteins [19], [20]. Rab6a and Rab6a' preferentially localize to the *trans*-Golgi cisterna/TGN where in their GTP-bound states they bind to a range of effectors. To date, more than 15 individual Rab6a and Rab6a' effectors have been identified [20], including motor proteins and members of the golgin family of coiled coil proteins. How Rab6a and Rab6a' functions are coordinated, with their overlapping roles and effectors is unknown. For simplicity, we will refer to Rab6a and Rab6a' collectively as Rab6.

Here, we investigated the role of Rab6 in regulating Golgi function for the trafficking and secretion of TNF in LPS-activated macrophages. Our findings serve to add Rab6 to the list of known molecular modulators of cytokine secretion and provide new insights about the role of Rab6 as a component of post-Golgi membrane carriers.

## Results

### Rab6 is Localized on Golgi Membranes and Maintains Golgi Stack Morphology in Macrophages

**Figure 1:**
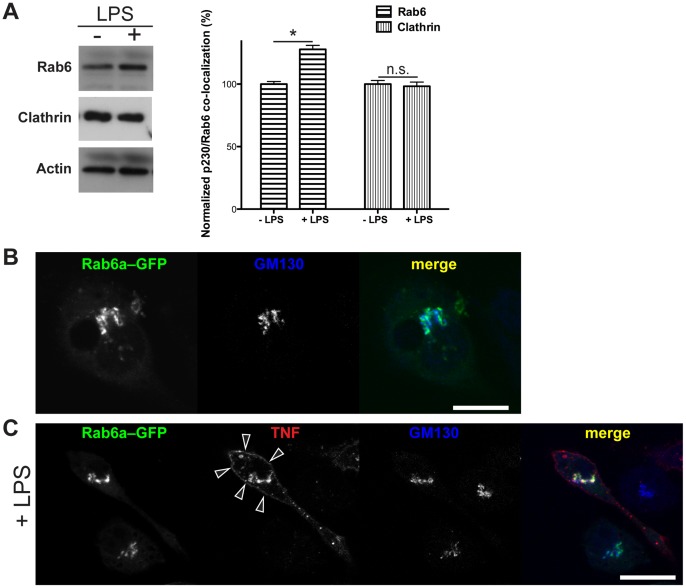
Rab6 localizes to Golgi membranes of RAW 264.7 macrophage cells. (A) After 2 h of LPS incubation significantly increased Rab6 protein level, while not affected clathrin, supporting a role for Rab6 in orchestrating TNF delivery form the TGN in a clathrin-independent manner. Rab6a–GFP localizes mainly on Golgi membranes (B) and its distribution is not perturbed by LPS treatment (C). LPS-activated cells clearly show a TNF surface staining (C, arrowheads). Original optical magnification 63X. Bar: 20 µm (A, B). * = p<0.05, n.s., not significant (pairwise comparisons).

Rab6 is typically localized on membranes of the Golgi complex (both *cis*- and *trans*-side) where it is involved in various trafficking steps (for a review see [21]). However, Rab6 has not previously been studied in macrophages where the Golgi complex, and the TGN in particular have crucial roles in cytokine trafficking and secretion. Previous work has shown Rab6 to be a regulator of secretory trafficking at the TGN [22], [23], [24], [25] and we became particularly interested in the role of Rab6 in inflammatory secretion in macrophages upon our finding shown in Figure 1A that Rab6 expression but not that of clathrin was induced by LPS. Endogenous Rab6 is expressed in macrophages and, interestingly, LPS induces an increased expression of Rab6 (Figure 1A) consistent with the up-regulation of other cell machinery, including several SNAREs that are required for cytokine secretion [7]. In contrast, Western blotting and quantification of clathrin heavy chain protein levels showed no significant increase in activated cells (Figure 1A). Thus, Rab6 is poised to have a role in Golgi trafficking in macrophages.

Transiently expressed Rab6a–GFP observed in the macrophage RAW 264.7 cell line shows typical labeling of the perinuclear Golgi complex and more diffuse staining in the cytoplasm (Figure 1B). LPS activates macrophages, initiating the synthesis of cytokines, chemokines and other secretory proteins [6]. Immuno-staining of LPS-activated macrophages typically shows bright staining of the newly synthesized *trans*-membrane precursor of the TNF in the Golgi complex (Figure 1C). TNF also passes through punctate peripheral compartments, shown previously to be recycling endosomes [26] (as seen in Figure 1C). Cleavage and release of TNF at the surface can be prevented with a TACE inhibitor (TAPI, TNF-alpha processing inhibitor) and then TNF delivered to the cell surface can be stained on fixed cells prior to cell permeabilization (Figure 1C, arrowheads). In LPS-activated cells, Rab6a–GFP remained localized on the Golgi complex where it overlapped with the TNF staining, but not with TNF in other compartments (Figure 1C). Identical results were observed in Rab6a'–GFP-transfected cells (data not shown).

### Dominant Negative Rab6 Mutants Limit TNF Trafficking

**Figure 2:**
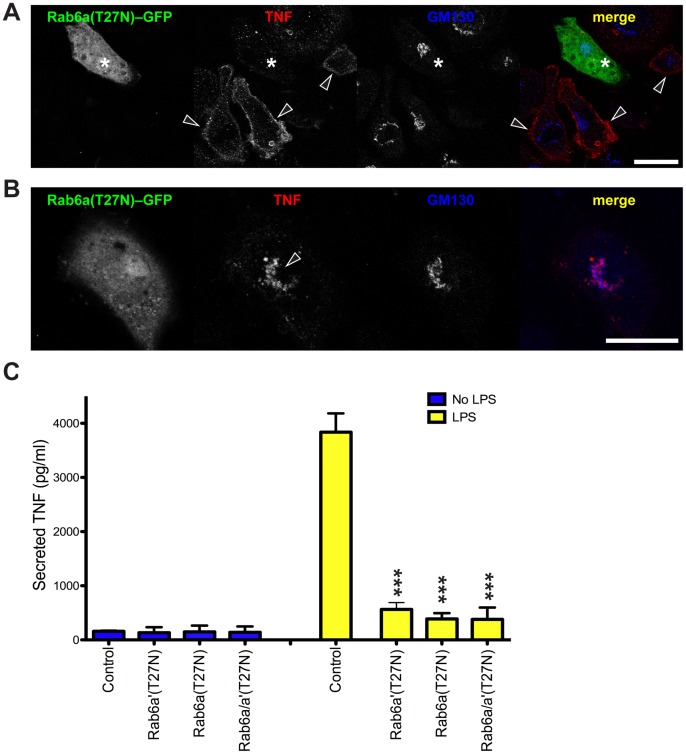
Expression of a single Rab6 inactive isoform inhibits TNF secretion. (A) After 2 h of LPS incubation Rab6a(T27N)–GFP transfected RAW 264.7 cells do not show plasma membrane staining for TNF (asterisks), while untransfected cells (arrowheads) clearly show surface staining indicative of TNF secretion. (B) Expression of Rab6a(T27N)–GFP results in accumulation of TNF in the Golgi complex. (C) Quantification of the TNF released in the growing medium per each experimental point has been graphed. Control is untransfected cells. Original optical magnification 63X (A, B). Bar: 15 µm (A, B). *** = p<0.001 (pairwise comparisons).

To explore whether Rab6 has a role in trafficking the secretory cargo TNF in macrophages, we used the transiently transfected cells over-expressing dominant negative (GDP-bound) Rab6 (T27N). Rab6a(T27N)–GFP typically appeared as diffuse staining throughout the cell rather than being directed to the Golgi membranes (Figure 2A), asterisks), as previously reported in other cells [27]. TNF surface staining was present on individual cells in LPS/TAPI treated cultures but not on the cells expressing Rab6a(T27N)–GFP, which had reduced or absent surface labeling of TNF (Figure 2A, arrowheads). This could be due to either reduced synthesis of TNF or perturbation in its trafficking and surface delivery. To test this, we stained fixed and permeabilized cells to detect intracellular TNF. The results show that inactive Rab6a does not impair the synthesis and the delivery of TNF to the Golgi complex (Figure 2B, arrowhead). We thus conclude that Golgi exit or post-Golgi trafficking of TNF is compromised when inactive Rab6a is overexpressed. Since, like other cells, macrophages have the additional Rab6a' isoform, we also expressed Rab6a'(T27N)–GFP and found that it too similarly reduced surface delivery and staining of TNF (data not shown).

To independently test TNF trafficking and release we collected cell supernatants from LPS-activated (2 hours) transfected cells for measurement of secreted TNF (Figure 2C). In control cells LPS induced robust release of TNF, however there was a similar and significant reduction (>80%) in TNF secretion from cells expressing either Rab6a(T27N)–GFP or Rab6a'(T27N)–GFP alone, or in combination (Figure 2C). Taken together these findings implicate Rab6 in the surface delivery and release of TNF after LPS stimulation. Furthermore, Rab6a and Rab6a' have overlapping or interchangeable roles in TNF secretion, consistent with previous studies in other systems which have shown that Rab6a and Rab6a' have similar, if non-totally overlapping, functions [28].

### Rab6 Depletion or Inactivation Blocks Delivery of TNF to the Cell Surface

**Figure 3:**
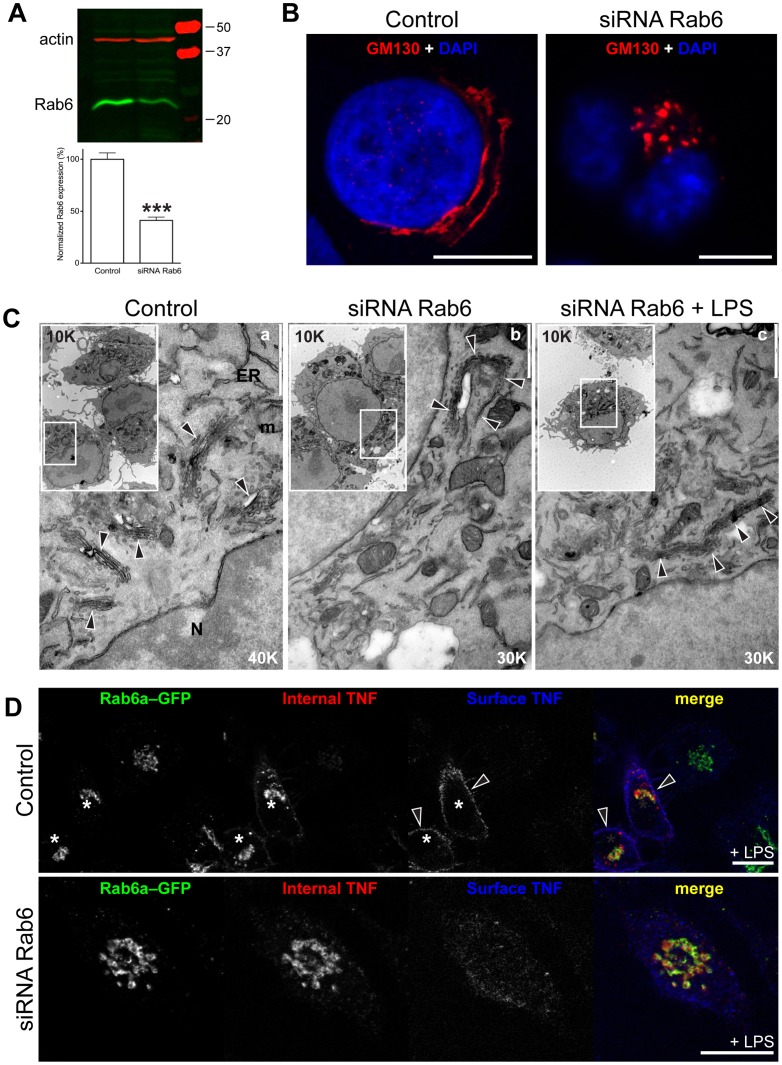
siRNA Rab6 affects Golgi morphology in RAW 264.7 macrophages. (A) Western blotting analysis confirmed a partial level of Rab6 depletion consisting of a drop of 40% in siRNA Rab6 macrophages, and (B) we also confirmed previous reports of collapsed, and frequently fragmented, Golgi complex compared with the most classical perinuclear shape distribution in control cells, using anti-GM130 as a Golgi marker. (C) Morphological changes on the Golgi complex in siRNA Rab6 macrophages LPS-activated for 2 h were visualized by EM; in control cells, the Golgi complex is formed by a multiple interconnected stacks (a, white arrowheads) approaching one to each other to form a ribbon. In siRNA Rab6 (b, white arrowheads) the Golgi complex consists of a huge single isolate ribbon, ticker and at least three times longer (white arrowheads), as previously characterized [25]. The addition of LPS did not significantly change the Golgi morphology (c, white arrowheads). Original magnification has been reported in each image (and relative inset), and the reference bars present on large images (vertically, top right). (D) After 2 h, LPS-activated RAW 264.7 cells clearly present internal and plasma membrane (surface) TNF staining (asterisks), while in siRNA Rab6 cells the TNF remains trapped in the cell. Original optical magnification 63X (B, D). Bars: 5 µm (B), 1 µm (C), 10 µm (D). N, nucleus; m, mitochondrion; ER, endoplasmic reticulum.

In order to further examine roles for Rab6 in trafficking, as our next approach we depleted Rab6 in macrophages using siRNA. We have previously shown that similar depletion of Rab6 in HeLa cells was signified by a distinctive rearrangement of the Golgi complex with an increase in number of Golgi cisternae and a *trans*-Golgi accumulation of COPI-coated vesicles [25]. Here, we selectively depleted Rab6 by siRNA as previously described [25], using a combination of specific murine oligonucleotides that disrupt both Rab6a and Rab6a' (see Materials and Methods). Western blot analysis confirmed the partial depletion of Rab6 protein to around 40% of control levels (Figure 3A). siRNA Rab6 in macrophages changed the normal Golgi morphology, consisting of a consolidate cluster in a perinuclear position (Figure 3B). After Rab6 depletion, at the light microscopy level, the Golgi complex was found to consist of a cluster of intensely labeled and sometimes fragmented ribbon (Figure 3B), as previously shown in HeLa cells [25]. At an ultrastructural level, the effects of Rab6 depletion were also evident in the altered morphology of the Golgi stacks (Figure 3C). Whereas in control cells we could easily distinguish single stacks that were patently arranged in clusters (Figure 3Ca, arrowheads), after siRNA Rab6 depletion, cells consistently had extended Golgi ribbons, three-four times the normal length and often contiguous in arrangement, with no individual stacks (Figure 3Cb, 3Cc, arrowheads) nor evident cisternal dilatation – again as previously described [25]. Importantly, the defective Golgi stack morphology persisted after LPS activation of the Rab6 depleted cells indicating that, at a time when the cells are producing cytokines, the Golgi morphology is still altered. These results demonstrate that depletion of Rab6 in macrophages has profound effects on Golgi stack morphology, consistent with proposed roles of Rab6 in protein trafficking and Golgi ribbon maintenance.

When RAW 264.7 cells were transiently transfected with siRNA Rab6, TNF was still present in the Golgi, despite the resulant and obvious changes in Golgi complex morphology in affected cells and significant reduction in TNF staining on the cell surface (Figure 3D, siRNA Rab6) compared to control cells (arrowheads in Figure 3D, Control). Of note, expression of the siRNA-resistant construct Rab6a–GFP (as well as Rab6a'–GFP; data not shown) in Rab6 depleted cells was not sufficient to recover the normal TNF trafficking (Figure 3D). In support of this observation is the above mentioned inhibition of a single Rab6 isoforms (by over-expressing Rab6a(T27N)–GFP or Rab6a'(T27N)–GFP) being sufficient to reduce the TNF secretion (Figure 2C). Together these results suggest a non-compensatory role of the two Rab6 isoforms (Figure 2C), with both required for TNF delivery to the plasma membrane and secretion. This also indicate that while TNF is successfully delivered to the Golgi under these conditions, intra- or post-Golgi trafficking might be compromised.

**Figure 4:**
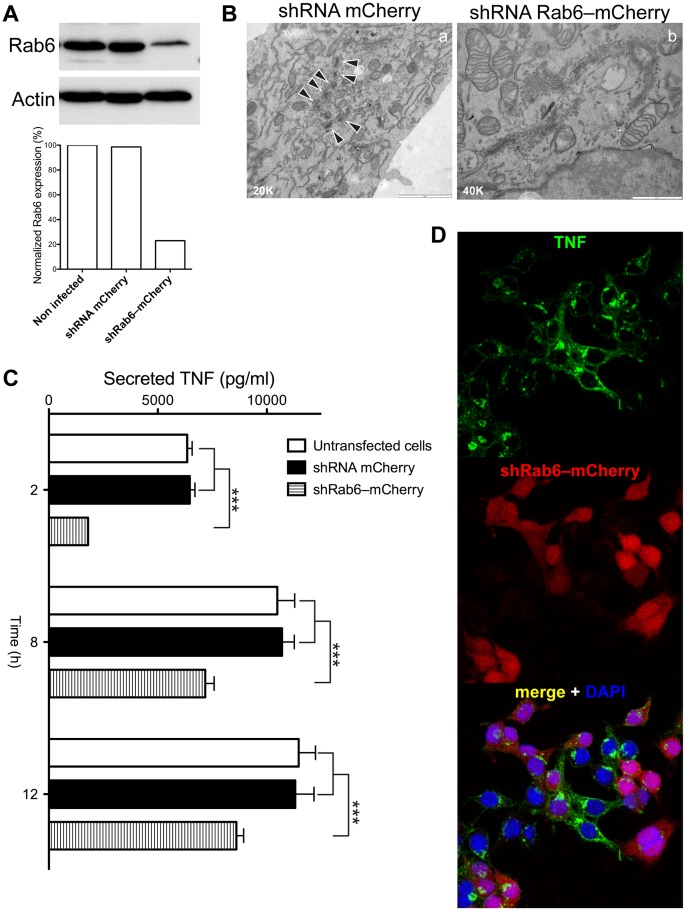
Stable depletion of Rab6 inhibits TNF release and significantly alters the Golgi morphology. (A) An shRNA Rab6–mCherry lentiviral construct expressed in RAW 264.7 cells induced a significant decrease in Rab6 expression detected by Western blotting analysis and the differences versus non-infected or infected with shRNA mCherry construct were graphed. (B) Morphological analysis confirmed a collapse of the Golgi complex in longer ribbon in shRab6 cells, frequently isolated from each other. Original magnification are indicated below left. Bars: 2 µm (shRNA mCherry), 1 µm (shRNA Rab6–mCherry). (C) Rab6 depletion caused a dramatic inhibition in TNF secretion after LPS activation in the initial 2 h, and recovered after longer experimental time points. **** = p<0.0001 (pairwise comparisons). (D) Dramatic inhibition of surface TNF expression was clearly evident in shRNA Rab6–mCherry expressing cells. Original optical magnification 63X. Bar: 20 µm.

In order to assess overall levels of TNF secretion we designed a small hairpin (sh) to deplete Rab6 in a more stable fashion. shRab6 tagged with mCherry (shRab6–mCherry) was produced as described in Materials and Methods, and the expressing cells were subsequently sorted by flow cytometry. Western blot analysis confirmed the efficiency of the shRab6 depletion of about 80% (Figure 4A). In these cells ultrastructural evaluation showed similarly altered Golgi morphology as observed in LPS-activated macrophages (Figure 4B), comparable with results obtained in siRNA Rab6, as described above (Figure 3C). The Golgi complex observed in the shRNA Rab6 cells (Figure 4Bb) is morphologically different from the stacked ribbon arrangement typical of control cells expressing shRNA mCherry (Figure 4Ba, arrowheads). Culture media of LPS-activated cells were collected over a time course to quantify TNF secretion. In untransfected cells or control cells expressing only shRNA mCherry, LPS induced a robust and increasing secretion of TNF over time. However, in shRab6–mCherry there was an initial reduction in TNF secretion (∼20% of normal level at 2 h). TNF secretion recovered over time, reaching ∼80% of normal levels by 12 h (Figure 4C). These results show that depletion of Rab6 causes a delay rather than a complete block in TNF secretion. Staining revealed that TNF was still present in the Golgi in cells expressing shRab6–mCherry and confirmed that its subsequent surface delivery was significantly reduced (Figure 4D). Thus, the delay in TNF secretion is likely to occur at the level of Golgi exit with reduced subsequent trafficking.

### Rab6-dependent Recruitment of p230/golgin-245/GOLGA4 onto Golgi Membranes

We next sought to investigate the specific transport step affected by Rab6 disruption. We have previously shown that TNF exits the TGN in dynamic tubular carriers demarked by the *trans*-golgin p230/golgin-245/GOLGA4 (hereafter p230) [8]. LPS up-regulates the formation of these carriers and depletion of p230 *in vitro* and *in vivo* impairs TNF trafficking out of the TGN, blocking its secretion [9]. Rab6 has also been found on TGN-derived carriers that mediate immediate post-Golgi transport [28] but these carriers have not been examined in macrophages or in the context of TNF secretion.

**Figure 5:**
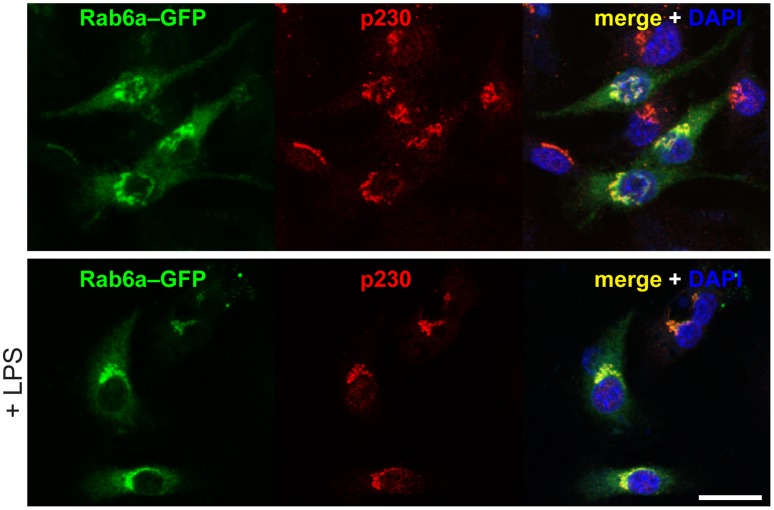
Rab6 localizes with p230 in the Golgi complex. RAW 264.7 macrophages were transfected with Rab6a–GFP, and fixed with 4% PFA in PBS for 30 min. We found a good co-localization level of Rab6a–GFP with the endogenous p230; LPS incubation for 2 h induced a significant increase of the co-localization of p230 on Rab6a-positive Golgi membranes. Original optical magnification 63X. Bar: 20 µm.

In cells before and after LPS activation, Rab6a–GFP was partially co-localized on the Golgi complex with endogenous p230 (Figure 5), as we have previously shown in HeLa cells [29]. Given the dynamic behavior of these TGN membranes and the proteins binding to them, two fixation conditions were initially compared: 4% paraformaldehyde (PFA; Figure 5) and cold methanol (Figure S1). Both methods yielded similar localization and overlap of these two proteins (Figure S1), and hereafter 4% PFA was used. Interestingly, following LPS activation there was a small but significant increase (∼25%) in the direct overlap of Rab6a–GFP and p230 when the rate of co-localization was measured (Figure 5; Figure S1). This may be suggestive of a rearrangement of TGN membranes induced by LPS that brings the two proteins onto the same membrane domains. Identical results were observed in Rab6a'–GFP transfected cells (data not shown).

**Figure 6:**
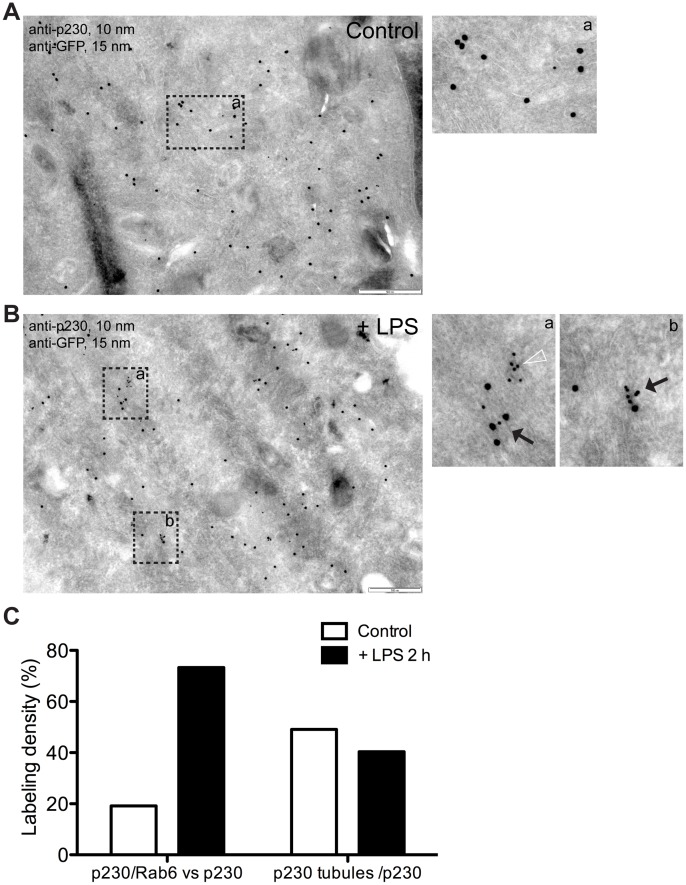
Cryo-immunogold EM reveals an increased co-localization of Rab6 and p230 in LPS-activated RAW 264.7 macrophages. Control (A) or LPS-activated (B) Rab6–GFP transfected RAW 264.7 macrophages were prepared for cryo-immunogold EM (see Materials and Methods) and then stained for GFP and p230. Unstimulated macrophages did not reveal Rab6 and p230 frequently associated on Golgi/TGN membranes (Aa), or clusters of p230. On the contrary, p230- (Ba) or p230/Rab6-positive membranes (Bb) are more evident in LPS-stimulated macrophages. (C) p230/Rab6-positive membranes increased more then two-fold when macrophages were LPS-activated (from 19.18% to 49.12%), while single p230 tubules decreased (from 73.29% to 40.35%). Bar: 500 nm (A, B).

Furthermore, we examined co-localization of p230 and Rab6 on Golgi membranes at the EM level in Rab6–GFP transfected macrophages using antibodies to immunogold label GFP and p230 (Figure 6). Prior to activation, labeling for both p230 and Rab6–GFP was seen on the same Golgi stacks but most often on separate substructures (Figure 6Aa). However, after LPS activation, the two proteins were more often co-localized with both gold labels found on the same membrane domains in Golgi stacks (Figures 6Ba, 6Bb, black arrows). After LPS activation gold label counting showed an increase in the proportion of p230 gold particles co-localized with Rab6 (Figures 6B, 6C). The number of identifiable membrane tubules immuno-labeled for p230 alone (Figure 6Ba, arrowhead) did not increase after activation implying that LPS-induced tubules are decorated by p230 and Rab6 together (Figures 6Ba, 6Bb, black arrows). Quantification of the labeling has been graphed (Figure 6C).

### p230 Localizes on Rab6-positive Vesicular/tubular Intermediates in the Golgi Complex

**Figure 7:**
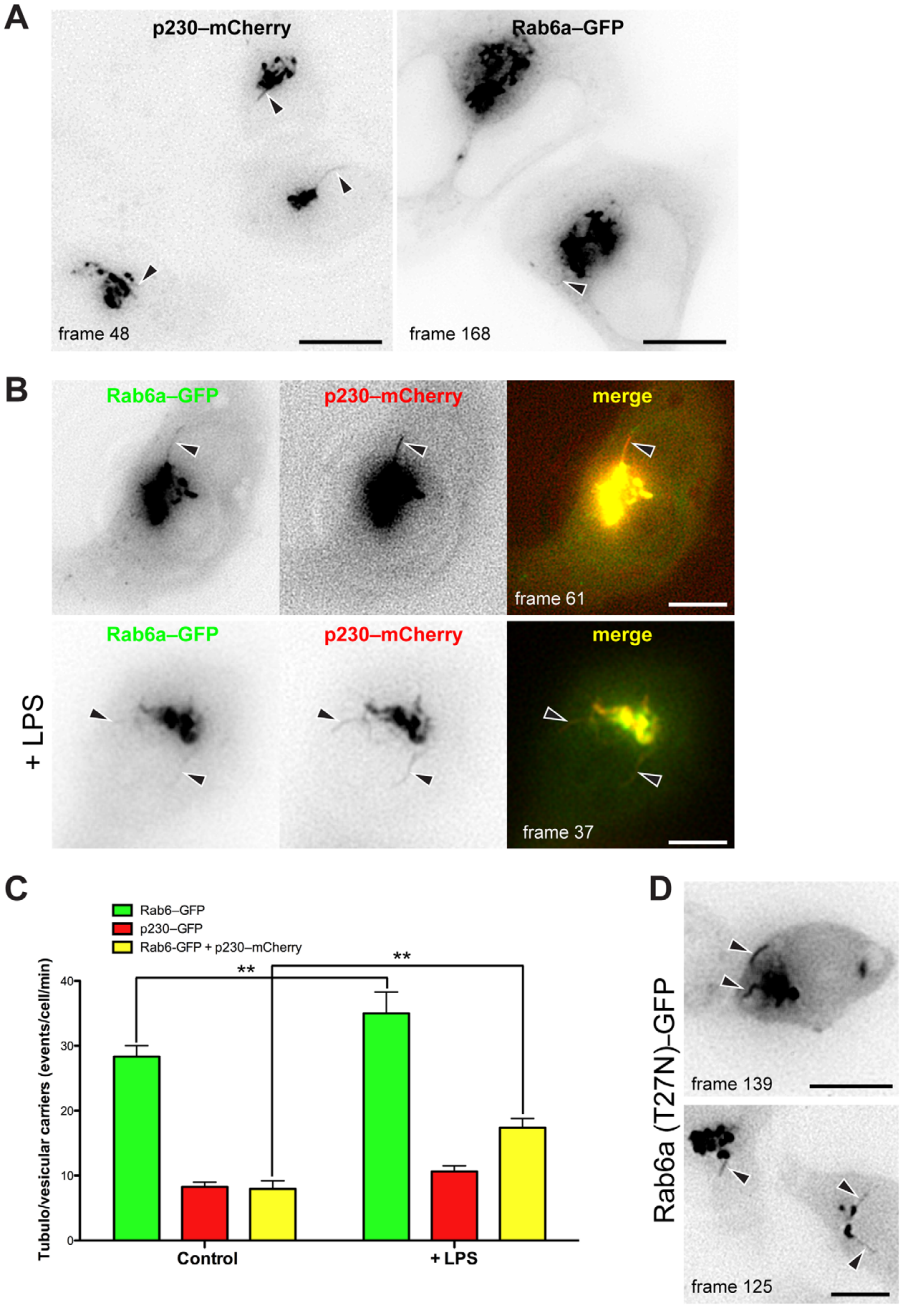
Rab6/p230-positive vesicular tubular carriers increase in LPS-activated RAW 264.7 macrophages. RAW 264.7 macrophages transiently expressing Rab6a–GFP (A, right), p230(GRIP)–mCherry (A, left), or co-transfected with Rab6a–GFP and p230(GRIP)–mCherry in control (B, upper panel) or LPS-activated RAW 264.7 macrophages (B, lower panel), were subjected to time-lapse recording. Singles frames derived from supplementary material Movies S1, S2, S3, and S4, respectively, are shown. (C) Quantification of tubule formation positive for Rab6, p230 or RAB6 and p230. Numbers of vesicles and tubules in single cells positive for Rab6-GFP, p230(GRIP)–mCherry and a combination of both that were formed *de novo* in control and LPS-activated RAW 264.7 macrophages from the Golgi complex within 300 s. The values represents means ± SD of eight cells observed for those expressing Rab6a–GFP (green), p230(GRIP)–mCherry (red), Rab6a–GFP and p230(GRIP)–mCherry (striped), in control or LPS-activated RAW264.7 macrophages. (D) Cells co-transfected with Rab6a(T27N)–GFP and p230(GRIP)–mCherry revealed a decreased level of p230-positive vesicular/tubular carriers. Asterisks highlight examples of tubules arising from the Golgi area. Original optical magnification 63X (A, B, D). Bars: 15 µm. ** = p<0.01 (pairwise comparisons).

We next investigated the dynamic distribution of Rab6 and p230 in living cells transfected with either p230(GRIP)–mCherry (hereafter p230–mCherry) or Rab6–GFP or with both probes (Figure 7). When p230–mCherry was expressed in macrophages and subjected to time-lapse recording, labeling was associated with Golgi membranes and it was found on dynamic tubules protruding from the TGN (Figure 7A, left, arrowheads; Movie S1). Rab6–GFP was also found associated with Golgi membranes and it was located on tubules that were more frequent by comparison (Figure 7A, right; Movie S2). When Rab6–GFP and p230–mCherry were co-expressed, they were co-localized on the Golgi membranes, and they appeared on a mixture of tubules - many positive for Rab6-GFP, some with p230–mCherry and some with both Rab6–GFP and p230–mCherry together (Figure 7B; Movie S3, arrowheads). Following LPS activation, tubules with both overlapping labels continued to appear and these were often configured with Rab6 on the tubule tips or more distally situated than p230, which was constrained nearer the base of the tubule (Figure 7B,+LPS; Movie S4). Quantification was achieved by counting vesicular and tubular intermediates formed *de novo* within a defined time interval in single cells. This revealed that Rab6–positive tubules were predominant and even more prevalent after LPS. More tubules labeled for p230 appeared after LPS stimulation, as we have previously shown [9]. However, here we now find that most of these LPS-induced tubules have both p230 and Rab6 associated with them (Figure 7C).

These observations suggest that Rab6 associates with vesicular and tubular carriers emerging constitutively from the TGN. Rab6 and p230 can appear on separate tubules or together on the same tubule. Interestingly, LPS promotes the formation of a class of Rab6/p230-positive vesicular/tubular carriers.

The expression of inactive Rab6 isoforms, Rab6a(T27N)–GFP (Figure 7D) and Rab6a'(T27N)–GFP (data not shown), dramatically reduced the frequency of the p230–mCherry tubules and inhibited their fission and release. This occurs in both control and LPS-activated cells (Movies S5 and S6, respectively). This finding indicates that one or other of the Rab6 isoforms is likely needed for release of TGN tubules, including those not obviously labeled with Rab6–GFP in the above recordings.

### Rab6 Stabilizes the Localization of p230 on the Golgi Complex

**Figure 8:**
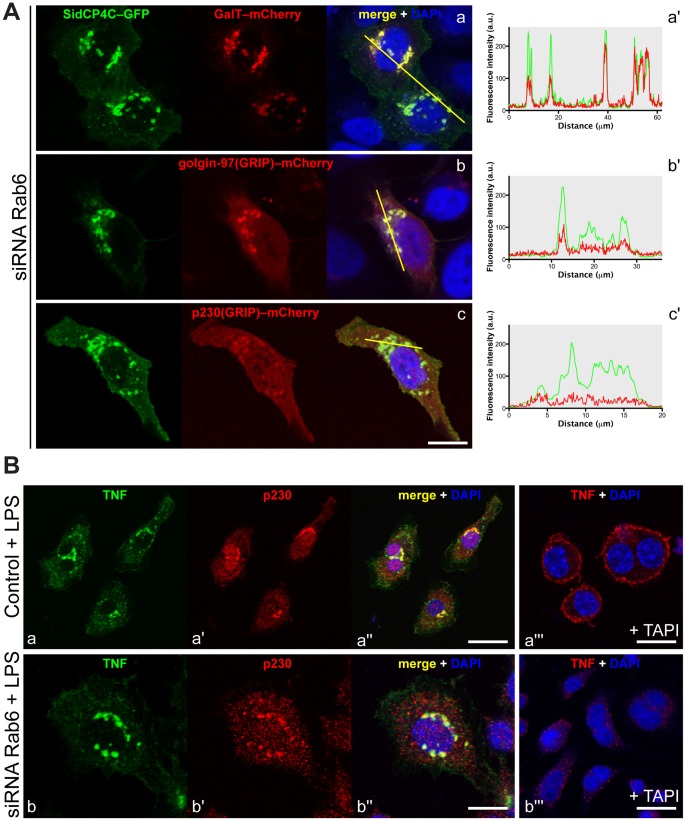
siRNA Rab6 affects the p230 localization on the Golgi membranes which is required for TNF secretion. (A) siRNA Rab6 RAW 264.7 macrophages were co-transfected with SidC_P4C_–GFP and GalT–mCherry (a), golgin-97(GRIP)–mCherry (b), p230(GRIP)–mCherry (c). Representative co-localization passing through the line scan (a-c), and plotted on the adjacent graphs (a'-c'), shows a clear decreased localization of p230 on the Golgi membranes (c'), less so efficient for golgin-97 (b'). (B) In the same experimental conditions, RAW 264.7 macrophages were stained for intracellular (a, b) and surface (a''', b''') TNF in the presence of LPS in control (a-a''') and in siRNA Rab6 (b-b''') cells. The addition of TAPI (a''', b''') was used to block TNF cleavage on plasma membrane, otherwise released into the growth medium, and to visualize the TNF staining on the surface of the cells. The depletion of Rab6 inhibits the arrival of TNF on the plasma membrane (b'''), which is concomitant with a partial redistribution of p230 (b'). Original optical magnification 63X. Bar: 10 µm (Aa-b, Ba''', Bb-b''), 15 µm (Bb'''), 20 µm (Ba–a'').

To directly determine if Rab6 addresses the Golgi localization of p230, we compared the p230 distribution with other Golgi markers in Rab6-depleted macrophages. The day after siRNA Rab6 depletion, RAW 264.7 macrophages were co-transfected with combinations of Golgi markers and golgin constructs: SidC_P4C_–GFP (a Golgi membrane protein), galactose-1-phosphate uridylyl transferase (GalT)–mCherry (a Golgi enzyme), golgin-97(GRIP)–mCherry (hereafter golgin-97–mCherry), p230–mCherry (Figure 8A). Co-expressed GalT–mCherry and SidC_P4C_–GFP were exactly colocalized denoting each of them as appropriate markers for the fragmented/collapsed Golgi complex in Rab6-depleted cells (Figure 8Aa). In the same Rab6-depleted cells golgin-97–GFP was found patently attached to Golgi membranes marked by SidC_P4C_–GFP (Figure 8Ab). On the contrary, under equivalent conditions, p230–mCherry labelling was significantly diminished over the Golgi area, again compared to SidC_P4C_–GFP and it appeared to be redistributed to the cytoplasm (Figure 8Ac). Line scanning of each representative image from each experimental condition provided informative redistribution over the Golgi area (Figures 8Aa'-c') Thus Rab6 depletion appears to diminish the binding of p230 to Golgi membranes, whereas golgin-97 binding appears much less affected. Quantification of Golgi marker co-localization confirms these observations (Figure S2A). Furthermore, cytosolic redistribution of both golgin-97 and p230 has been quantified (Figure S2B), confirming that loss of Rab6 and disruption of the Golgi are associated with a significant redistribution of p230 in the cytosol, less so for golgin-97.

TNF and p230 labeling were also compared in LPS-activated cells. In control cells, TNF and p230 co-localized over the Golgi (Figures 8Ba–a'') and TNF was delivered to the cell surface (Figure 8Ba'''). In Rab6-depleted cells, TNF was present in the Golgi (Figures 8Bb–b''), but reduced on the cell surface (Figure 8Bb''') and p230 labeling was also diminished over the Golgi, with more in the cytoplasm (Figure 8Bb'). Quantification of the p230 redistribution is also presented (Figure S2C). Taken together these results suggest that p230 recruitment to the Golgi membranes is compromised by depletion of Rab6 and this occurs alongside the impaired trafficking of TNF.

We also characterized the p230 redistribution in LPS-activated macrophages expressing the inactive Rab6 mutants (Figure S3). Expression of Rab6a(T27N)–GFP or Rab6a'(T27N)–GFP alone did not disrupt the Golgi localization of p230–mCherry, suggesting that neither of the isoforms on its own can disrupt p230 or p230 target membranes (Figure S3). Since the siRNA Rab6, which depletes both isoforms induces a cytosolic redistribution of p230, while over-expression of single mutants had no effect, which could suggest that optimal p230 Golgi recruitment (or stabilization) requires both Rab6 isoforms functioning in redundant roles.

### Brefeldin A Induces Faster p230 Redistribution from Golgi Membranes than Rab6

**Figure 9:**
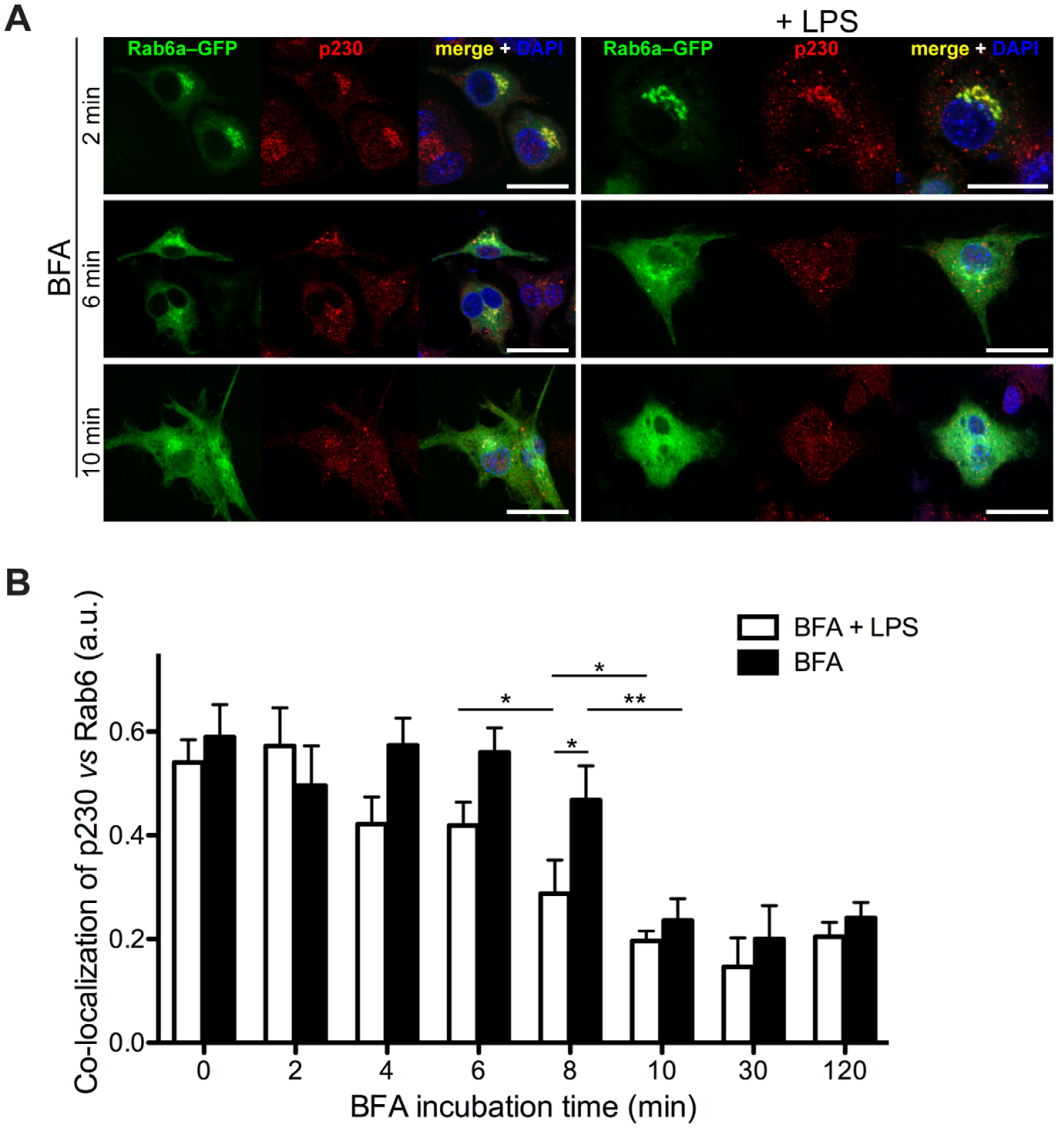
Rab6 and p230 show different BFA-induced cytosolic redistributions. RAW 264.7 macrophages transftected with Rab6a–GFP were incubated over a time course with BFA 5 µg/ml to follow the cytosolic redistribution of both Rab6 and p230 in control (A) or LPS-activated (B) cells. p230 redistributed faster on LPS-activated macrophages where TNF production had been stimulated, while Rab6a redistribution was not affected by the presence of LPS. These changes, visualized as p230/Rab6 ratios, and relative statistical differences have been plotted (C). Original optical magnification 63X (A, B). Bar: 25 µm (A, B). * = p<0.05, ** = p<0.01 (pairwise comparisons).

Finally, p230 recruitment to Golgi membranes was investigated in the presence of brefeldin A (BFA). BFA is an inhibitor of guanine nucleotide exchange factor for Arfs; treating cells with this drug causes dissociation of not only Arf itself but also effector coat proteins, such as COPI and AP-1 complexes, from Golgi membranes [30], [31]. Cells were transfected with Rab6–GFP and incubated over a time course with BFA. Binding of p230 to Golgi membranes is BFA sensitive and here we show p230 immuno-staining over the Golgi; by 6 min with BFA, p230 was redistributing to the cytoplasm and by 10 min it was depleted from the Golgi (Figure 9A). By comparison, over the same time period Rab6a–GFP showed slower redistribution. p230 redistribution on the Golgi membranes over a BFA incubation time course (with or without LPS) has been graphed including additional experimental time points (Figure 9B). Identical results were observed in Rab6a'–GFP transfected cells (data not shown). These preliminary results suggest that p230 and Rab6 are recruited/maintained independently of each other on the Golgi membranes. Rab6 would be in a position to stabilize p230 binding and this is one mechanism we propose for Rab6 in supporting the trafficking and secretion of TNF, not excluding that the effect of Rab6 could be indirect and mediated by Rab6 effectors.

## Discussion

The intracellular transport of TNF and other cytokines through the constitutive secretory pathway in macrophages necessarily involves the Golgi complex as a major compartment for posttranslational modification and trafficking. The *trans*-Golgi and the TGN are well known sorting stations [32], [33] where the post-Golgi transport and fate of cytokines are largely determined. Rab GTPases associated with the Golgi complex are thus poised to have major roles in this crucial secretory pathway during macrophage immune responses. Thus cued, we investigated possible roles for Golgi-associated Rab6 in Golgi maintenance and TNF trafficking in macrophages.

GDP-locked mutants of Rab6a and Rab6a' (T27N) demonstrably reduced or perturbed the accumulation of TNF at the cell surface and its short term release, whilst newly synthesized TNF was still present in the Golgi complex. This indicated a likely requirement for active Rab6a and/or Rab6a' for the trafficking of TNF between the Golgi and the cell surface. Indeed, it has been shown that Rab6(T27N) inhibits anterograde Golgi trafficking in other cells, including both intra-Golgi and post-Golgi trafficking steps [25], [34].

Depletion of Rab6 protein from cells with siRNA or shRNA, which evidently included loss of both Rab6a and Rab6a' isoforms, had a dramatic effect on Golgi structure, causing the running together of Golgi ribbons previously visualized by tomography [25]. This indicates that Rab6 has a role in maintaining Golgi stack and ribbon structure in macrophages, similar to that in other cells. Interestingly, whilst the macrophages reflected the cisternal changes seen previously, the accumulation of vesicles caused by Rab6 depletion in HeLa cells [25] was not so evident here. This may signify that the dynamic tubules seen in live cell imaging (Figure 7 and Movies S1, S2, S3, S4, S5, S6), more so than vesicles, are the major carrier type for Golgi-associated transport in macrophages. Nonetheless, since macrophages were able to grow and to respond to activators like LPS, these structural changes to the Golgi are not fatally detrimental to cell survival. Notably the over-expression of each of the Rab6–GDP mutants did not cause a similar change to Golgi structure, most likely because the underlying endogenous forms of Rab6 were still present for Golgi maintenance, or due to the functional presence of the other Rab6 isoforms.

Upon Rab6 depletion, the secretion of TNF was reduced. Over a time course it was evident that Rab6 depletion most severely reduced the initial release of TNF, and then secretion levels recovered somewhat at later times. This recovery may be due to the LPS-induced expression of Rab6 identified here by Western blotting (since the siRNAs were not fully penetrable) or by some other compensatory effect. Nevertheless it appears that the Golgi cisternal dysmorphology resulting from Rab6 depletion does not itself prevent the transit of membrane-bound cargoes like TNF. The role of Rab6 in the regulation of membrane trafficking and the maintenance of Golgi organization has been recently reviewed [35], supporting a role for the Golgi maintenance operated by Rab6 effector proteins which is critical for correct intra- and post-Golgi membrane trafficking. Our results from both Rab6 depletion and over-expression of the dominant negative mutants show perturbation and reduction of cell surface delivery of TNF, in keeping with previous Rab6 roles noted in anterograde transport [22], [23], [24], [34]. While TNF trafficking is clearly inhibited at the level of the Golgi and post-Golgi transport is affected after manipulation of Rab6, we did not attempt to map intra-Golgi transport of TNF in these experiments. In this and previous studies [3], [6] we have recorded that perturbing post-Golgi transport does not cause a dramatic accumulation of TNF in the Golgi, as seen for exogenous cargo [25], and this is likely because the synthesis of endogenous TNF is tempered by the trafficking block.

Whereas the recycling endosomes dictate the final steps in the delivery of TNF and some other cytokines to the cell surface for release [3], [4], [11], [26], TGN-derived carriers have emerged as sites for discriminating regulation of TNF trafficking by multiple families of trafficking proteins, including SNAREs, golgins and PI3Kδ [36], [37]. At the TGN, the trans-membrane TNF is sorted and loaded into tubular carriers labeled with the golgin p230 [9] for transport to recycling endosomes. p230 is one of the so-called trans-golgins [38] which have roles in trafficking as well as in Golgi maintenance [39], [40], [41], [42], [43], [44]. In the present study we provide evidence that both Rab6 and p230 regulate TNF trafficking and may also both regulate Golgi maintenance – at least at the level of TGN carriers. LPS enhanced the Rab6 and p230 labeling together and in live cells we showed that TGN-derived carriers most often have both p230 and Rab6 on the same tubules, albeit on only partially overlapping domains. While the recruitment of p230 to these tubules is well established, the precise role of p230 on the carriers is not well understood. Rab6 too is a well-known component of Golgi-derived tubules that transport cargo to recycling endosomes or on carriers moving to the cell surface where it has multiple reported roles including (*i*) vesicle/tubule fission from the Golgi orchestrating cargo exit from the Golgi/TGN through one of its effectors, myosin II [23], (*ii)* fission of one population of carriers [45] and (*iii*) in docking and fusion of carriers at the cell surface [22], [24].

In macrophages we found Rab6 constitutively bound to many carrier tubules emerging from the TGN, consistent with its association on multiple types of carriers for post-Golgi transport. p230 was on a more specific subset of tubules, including those transporting TNF [9], many of which we now show also have Rab6 as a tubule component. Rab6 was often distally located on the tubules and this, together with its widespread distribution on tubules, are consistent with it having a necessary mechanical role in TGN exit. On this basis we would predict that Rab6 is also required for the TGN exit of other, soluble cytokines, like IL-6 and IL-10 that use additional carriers to those used for TNF transport [46]. Moreover it is likely that Rab6 has a general and necessary role for post-Golgi transport in macrophages and in this guise its activity would affect many of the dynamic trafficking pathways that are needed for macrophage immune functions. Our observations on Rab6 localization and behavior on TGN tubules in live macrophages, concur with earlier studies in HeLa cells showing Rab6 participation in the processivity of these carriers [22], [24].

Depletion of Rab6 caused a significant cytosolic redistribution of p230, seemingly reducing the binding of p230, but not of golgin-97 to Golgi membranes. This accompanied the impairment of TNF transport and is thus a possible mechanism to explain this trafficking defect. *A priori* this might also implicate Rab6 in selective, direct recruitment of p230 to the tubule membranes. p230 and golgin-97, both localizing at the TGN but in different membrane domains [8], interact with Arl1 through their GRIP domain [47], [48], [49], [50] and are significantly redistributed in siRNA Arl1 cells [29], [51]. Furthermore, the membrane attachment of Rab6 and p230 was then compared over a time course of BFA treatment, which clearly showed a discordant and sequential displacement of p230 followed significantly later by Rab6. We thus conclude that the Arl1-dependent binding of p230 on the TGN, which is required to form the tubules that orchestrate TNF transport, is most likely stabilized rather than being initiated by Rab6. On the contrary, Rab6 is not necessary to recruit other *trans*-golgins like GCC185 [29], highlighting a somewhat specific role for Rab6 in regulating/stabilizing the p230 recruitment for TNF secretion in macrophages. Of relevance is that Rab binding sites of GRIP domain proteins have been mapped and the Rab binding of Drosophila p230 did not include Rab6 [52]. Hence, the affect of Rab6 on p230 may be mediated indirectly via Rab6 effectors. As a further note, our results suggest that p230 stabilization relies on both Rab6a and Rab6a', whereas inactive mutants of only one or other isoforms were sufficient to inhibit TNF delivery to the plasma membrane. Selective RNAi knockdown of each isoform in the future would complement the results from the dominant negative mutants and serve to confirm the need for both Rab6a and Rab6a' in this role.

In conclusion we have shown that Rab6 has essential roles in maintaining Golgi morphology and secretory trafficking in activated macrophages. One specific function for Rab6 is demonstrated by its requirement for the efficient delivery of TNF to the cell surface and for secretion in LPS-activated macrophages.

Rab6 is part of a Rab cascade in the Golgi along with Rab33 and associated GEFs, Ric1p and Rgp1p to regulate both anterograde and retrograde transport through the Golgi [53], [54]. Moreover, Varp (a Rab21 GEF) interacts with p230 and Rab21 during secretory vescicle exocytosis, and in turn the GTP-bound Rab21 intracts with microtubule and actin cross-linking factor 1, an actin and microtubule regulator and a binding partner of p230 [55], [56]. It will therefore be of interest to examine additional proteins in this cascade in macrophages. A role for Rab6 in TNF trafficking, as shown in live cells, is the mediation of TGN-derived tubular carriers for post-Golgi transport of TNF [9]. Another component of these carriers is the golgin p230, necessary for TNF transport and the stabilization of p230 on these membranes is proposed as one mechanism for Rab6 regulation of TNF trafficking.

## Materials and Methods

### Antibodies

We used monoclonal antibodies to GM130 and golgin-97, and rat anti-TNF for intracellular staining (BD Biosciences), polyclonal anti-TNF for surface staining (Calbiochem). The following were also used: nanogold-conjugated Fab fragments of anti-rabbit immunoglobulin G (IgG); protein A conjugated with colloidal gold, 10 or 15 nm (Dr J. Slot, Utrecht University, Utrecht, Netherlands); anti-rabbit, anti-mouse and anti-rat antibodies conjugated with Alexa 488, Alexa 555 and Alexa 633 (1∶200–500; Molecular Probes). To generate an anti-mouse p230 antibody, we used a synthetic peptide corresponding to the N-terminal 34 residues of mouse p230 (p230_1–34_), with an additional cysteine residue at the C-terminus (Research Transfer Facility, BIO21 Institute). The sequence of peptide is: MFKKLKQKISEEQQQLQQALAPAQASSSSSTPTRC. The p230_1–34_ was directly injected subcutaneously into a rabbit and three additional boost injections were given 28, 56 and 91 days after the initial immunization followed by a terminal bleed at 103 days; the immunization as well as the bleeds were performed commercially by WEHI Antibody Facility (Australia). Antibodies were affinity-purified using a column of p230_1–34_ peptide conjugated to SulfoLink Coupling Gel (Pierce). The affinity-purified antibodies were demonstrated to be specific for p230 as (i) they detected perinuclear Golgi structures in mouse NIH3T3 cells, RAW 264.7 and bone marrow-derived macrophages by indirect immunofluorescence, whereas no reactivity was observed in cells depleted of endogenous p230 and (ii) they immunoprecitated a protein ∼230 kDa, corresponding to the size of endogenous p230.

### Constructs

Previously described GFP–Rab6a and GFP–Rab6a' [27], and the dominant negative mutants Rab6a(T27N)–GFP and Rab6a'(T27N)–GFP [34] were kindly provided by Prof. Bruno Goud (Institut Curie, Paris, France). GalT–mCherry was kindly provided by Prof. Alan S. Verkman (University of California - San Francisco, CA, USA) and generated as previously reported [57]. GT–GFP was a gift from Prof. Jennifer Lippincott-Schwartz (National Institutes of Health, MD, USA). Plasmids encoding for the following chimeric proteins have been described previously: GFP–tagged C-terminal GRIP domains of p230 [p230(GRIP)–GFP] and golgin-97 [golgin-97(GRIP)–GFP] [58], [59], [60], and TNF–mCherry [11]. The GRIP domain of golgin-97 and p230 were also sub-cloned into the pmCherry-C1 vector (Clontech). The minimal binding domain for phosphatidylinositol-4-phosphate from the *Legionella pneumophila* effector SidC (amino acids 609–776, SidC_P4C_) [61] was cloned by PCR into the pEGFP-C1 vector (Clontech) [62].

### Reagents

We used LPS from *Salmonella enterica* serovar Minnesota Re595, BFA, and DMSO (Sigma-Aldrich), and TAPI (Santa Cruz Biotechnology).

### Cell Culture, Transfections, siRNA and shRNA Knockdowns

The RAW 264.7 murine macrophage cell line (ATCC, TIB-71) was cultured in RPMI 1640 medium (Lonza) supplemented with 10% heat-inactivated FCS (Thermo Trace) and L-glutamine 2 mM (Invitrogen) in humidified 5% CO_2_ at 37°C as previously described [63]. For transient expression of cDNA, cells at 50% confluency were transfected using Lipofectamine 2000 (Invitrogen) according to the manufacturer’s instructions. Cells were typically used for experiments 6–24 h after transfection. Where indicated, cells were activated by priming with 100 ng/ml LPS for 2 h. For siRNA Rab6 cells were transfected with specific stealth siRNAs against Rab6 (Invitrogen), cultured for 24 h, re-transfected under the same conditions and cultured for another 24 h before LPS activation.

A lentivirus-based shRNA system [64], [65] was used to deplete Rab6 in RAW 264.7 cells. The lentivirus expression vector LentiLox pLL5.0 (backbone pLL3.7) was a gift from J. Bear (UNC Chapel Hill, School of Medicine, NC, USA) [65], [66]. pLL5.0 is a lentiviral expression plasmid used to drive the expression of the mCherry-tagged shRNA sequence [67]. Algorithms from Dharmacon were used to predict the sequences that would lead to silencing of mouse Rab6 (NM_001163663.1). The predicted sequence was used to design an shRNA containing a stem loop sequence based on previous studies [68], and these were cloned into pLL5.0, thus yielding pLL5.0 shRab6–mCherry. In brief, the PLL5.0 shRab6–mCherry vector was obtained by inserting into the PLL5.0 vector a 55 bp sequence containing the transcriptional start site for U6 promoter, a 19 bp sequence derived from the mouse Rab6 target transcript (5'-TGATCACCCGATTCATGTA-3', corresponding to nucleotides 724–742 of the Rab6 coding sequence) separated by a short spacer (5^′^-TTCAAGAGA-3^′^ ) from the reverse complement of the same 19-nt sequence, and the transcriptional termination site for the RNA polymerase. This construct was used to generate a lentivirus expressing shRab6–mCherry according to a well established protocol [69]. All cells that were mCherry-positive also express the shRNA Rab6. The efficiency and reproducibility of Rab6 silencing was also confirmed by Western blotting (see below). Single-cell suspensions were sorted by flow cytometry according to high levels of reporter gene expression. Of note, after sorting all of the cells showed clear shRab6–mCherry expression, a phenotype which was non-lethal throughout experimental time points. An empty lentiviral mCherry vector was used as a control.

### Cytokine Secretion Assay

RAW 264.7 macrophages were transfected with the Rab6 dominant negative constructs and/or incubated in the presence of LPS 100 ng/ml, BFA 5 µg/ml for 2 h. Levels of secreted TNF in the culture medium were then quantified using a mouse TNF Elisa kit II (BD OptEIA) according to the manufacturer’s protocols.

### Immunofluorescence Microscopy and Live Cell Imaging

Immunofluorescence staining was performed as previously described [5] on coverslip-adherent macrophages fixed for 30 min in 4% PFA, quenched with glycine 20 mM for 10 min prior to permeabilization with saponin-containing blocking solution (0.1% saponin, 0.5% BSA, 0.1% fish skin gelatin, 0.02% NaN_3_, NH_4_Cl 50 mM in PBS) for 30 min, and then incubated with appropriate primary and secondary antibodies. Nuclei were counterstained with DAPI. Coverslips were mounted in ProLong Gold reagent (Invitrogen) prior to imaging. For live cell experiments, RAW 264.7 macrophages were cultured on 35 mm glass-bottom dishes (MatTek). Confocal microscopy on fixed cells was performed using a Leica TCS SP2 imaging system.

Live cell imaging was performed using a Personal DeltaVision Olympus IX71 inverted widefield deconvolution microscope equipped with an Olympus Plan apochromat 60× NA 1.35 oil objective and a 37°C incubator. Images were captured using a Roper CoolSNAP HQ2 monochrome camera. Imaging analysis of all other data was performed using ImageJ software (version 1.43; National Institutes of Health).

### Preparation of Cell Lysates, SDS-PAGE, and Western Blotting

Cells were washed three times in ice-cold PBS and then lysed in sodium chloride 150 mM, Tris-HCl 50 mM pH 7.5, 1% Igepal (nonidet-P40, Sigma). Protein concentrations were determined by a BCA assay-(bicinchoninic acid protein) (**Pierce Chemical**), and equal amounts of proteins were resolved on SDS-PAGE gels, transferred onto PVDF membrane (Immobilon-FL, Millipore, or PVDF BioTrace PVDF Pall) immunoblotted and detected with either enhanced chemiluminescence system (PerkinElmer Life Sciences) or Odyssey® infrared imaging detection (LI-COR® Biosciences). The ECL system utilized Biotrace- PVDF and HRP-conjugated secondary antibodies. Odyssey system required Immobilon-FL membrane and secondary antibodies coupled to IRDye® 800 or IRDye® 680 fluorophores.

### Electron Microscopy

The procedures used to prepare mammalian cells for electron microscopy imaging have been previously described [25], [70]. Briefly, thin (70 nm) sections cut with UltraCut-UCT (Leica) microtome were collected onto Formvar-coated copper slot grids and post-stained [with aqueous uranyl acetate or Reynold’s lead citrate (Electron Microscopy Sciences)] to enhance contrast/visualization. Thin sections were surveyed using a JEOL 1011 electron microscope (JEOL Australasia Pty Ltd) operated at 80 kV to assess the quality of ultrastructural preservation, collect sets of 2D images depicting representative regions in both Rab6-depleted and control cells, on correlated light microscope images of the same grids before embedding. Cryo-immunogold EM was performed exactly as previously described [71].

### Statistical Analyses

Statistical analysis was performed using two-tailed Student’s *t*-test or two-way ANOVA followed by Bonferroni post-testing using Prism software (version 5; GraphPad Software Inc.). A value of p<0.05 was considered significant.

## Supporting Information

Figure S1 **Rab6/p230 co-localization ratio on Golgi membranes is not affected by different fixatives.** No difference in the co-localization level of Rab6–GFP with endogenous p230 was observed in RAW 264.7 macrophages fixed with cold methanol for 10 min at 4°C *versus* 4% PFA fixed macrophages. LPS incubation for 2 h induced a significantly increased co-localization of p230 on Rab6-positive Golgi membranes. Normalized co-localization levels are plotted in the graph. Original optical magnification 63X (A). Bar: 20 µm (A). ** = p<0.01 (pairwise comparisons).
(TIF)

Figure S2 **Quantification of golgin redistributions in siRNA Rab6 cells.** (A) Co-localization of p230–mCherry and golgin-97–mCherry in the experimental conditions summarized in Figure 8 was normalized to the SidC_P4P_–GFP used as a Golgi marker. (B) Golgi localizing p230–mCherry and golgin-97–mCherry was normalized to the cytosolic redistribution. (C) Quantification of p230 redistribution in control and siRNA Rab6 LPS-activated cells as a ratio of Golgi complex and cytosolic area. * = p<0.05, *** = p<0.001 (pairwise comparisons).
(TIF)

Figure S3 Expression of Rab6a(T27N)–GFP and Rab6a'(T27N)–GFP inhibit Golgi-to-plasma membrane TNF trafficking, but not the Golgi localization of p230. Inactivation of single Rab6a or Rab6a' isoforms by over-expressing the GFP–tagged dominant negative (T27N) proteins resulted in an inefficient cytosolic redistribution of p230, which was for siRNA Rab6. Original optical magnification 63X. Bar: 10 µm.
(TIF)

Movie S1 **RAW 264.7 macrophages were transfected with p230(GRIP)–mCherry.** Images were acquired using a DeltaVision microscope for 5 min at 0.5 s intervals.
(AVI)

Movie S2 **RAW 264.7 macrophages were transfected with Rab6a–GFP.** Images were acquired using a DeltaVision microscope for 5 min at 0.5 s intervals.
(AVI)

Movie S3 **RAW 264.7 macrophages were transfected with Rab6a–GFP and p230–mCherry.** Images were acquired using a DeltaVision microscope for 5 min at 0.5 s intervals.
(AVI)

Movie S4 **RAW 264.7 macrophages were transfected with Rab6a–GFP and p230–mCherry and stimulated for 2 h with LPS before recording.** Images were acquired using a DeltaVision microscope for 5 min at 0.5 s intervals.
(AVI)

Movie S5 **RAW 264.7 macrophages were transfected with Rab6a(T27N)–GFP.** Images were acquired using a DeltaVision microscope for 5 min at 0.5 s intervals.
(AVI)

Movie S6 **RAW 264.7 macrophages were transfected with Rab6a(T27N)–GFP and p230–mCherry, and stimulated for 2 h with LPS before recording.** Images were acquired using a DeltaVision microscope for 5 min at 0.5 s intervals.
(AVI)
