# Supplementary figures and images for: Rab6a/a’ Are Important Golgi Regulators of Pro-Inflammatory TNF Secretion in Macrophages
Source: PLoS One. 2013 Feb 21;8(2):e57034. doi: 10.1371/journal.pone.0057034 (PMC3578815; doi:10.1371/journal.pone.0057034)

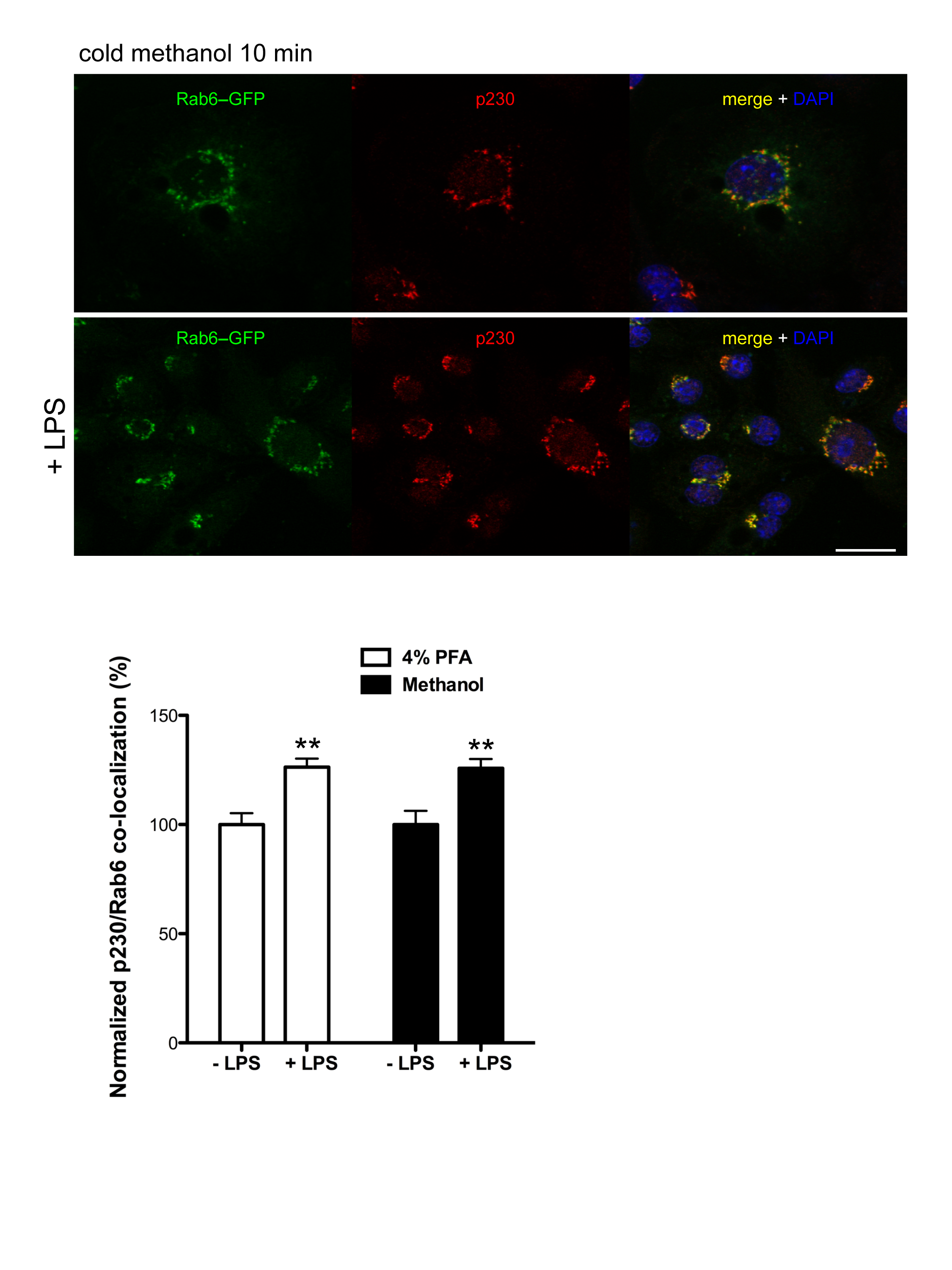

Supplement: Figure S1 — Rab6/p230 co-localization ratio on Golgi membranes is not affected by different fixatives. No difference in the co-localization level of Rab6–GFP with endogenous p230 was observed in RAW 264.7 macrophages fixed with cold methanol for 10 min at 4°C versus 4% PFA fixed macrophages. LPS incubation for 2 h induced a significantly increased co-localization of p230 on Rab6-positive Golgi membranes. Normalized co-localization levels are plotted in the graph. Original optical magnification 63X (A). Bar: 20 µm (A). ** = p<0.01 (pairwise comparisons). (TIF) [file pone.0057034.s001.tif]

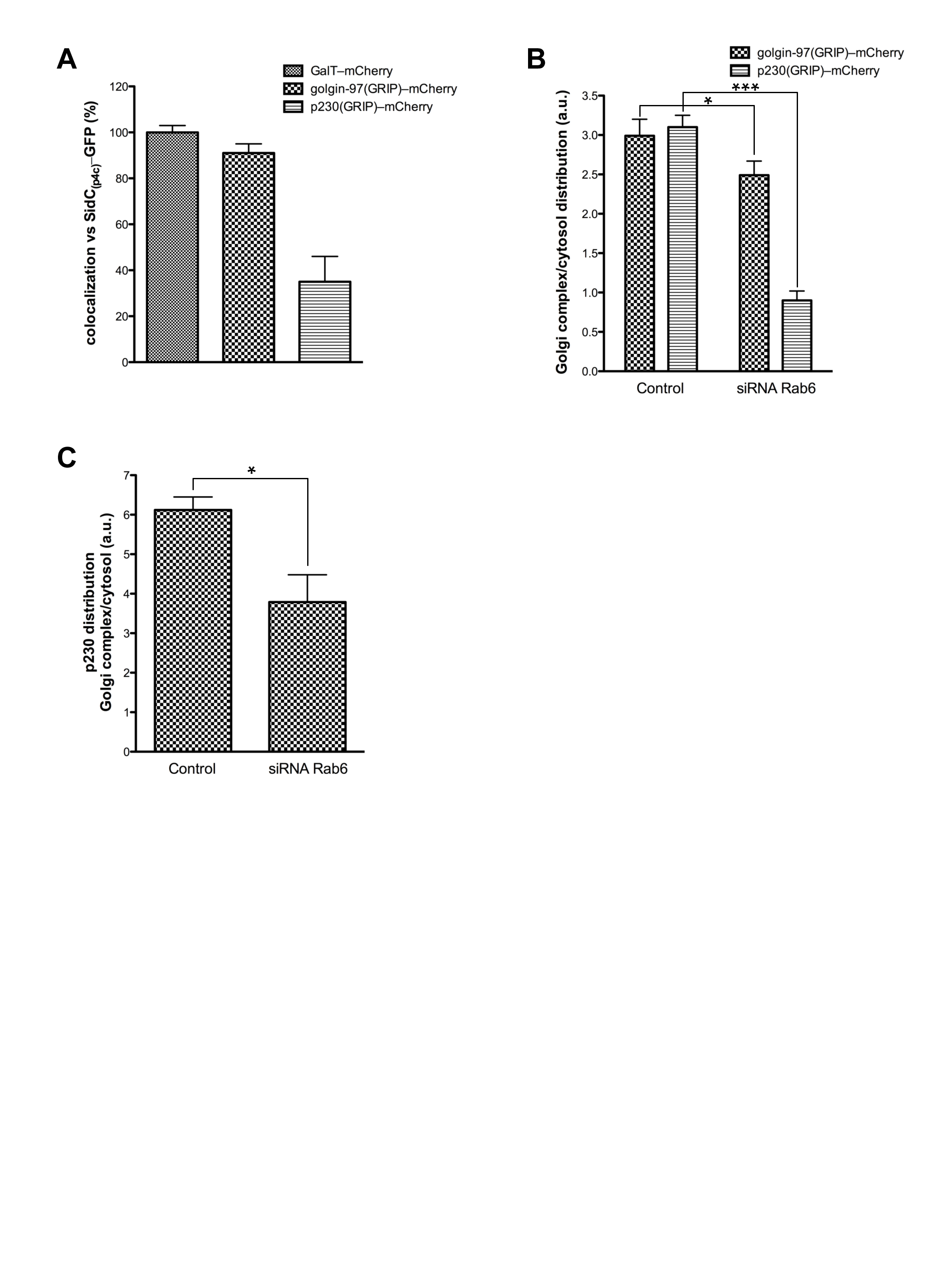

Supplement: Figure S2 — Quantification of golgin redistributions in siRNA Rab6 cells. (A) Co-localization of p230–mCherry and golgin-97–mCherry in the experimental conditions summarized in Figure 8 was normalized to the SidCP4P–GFP used as a Golgi marker. (B) Golgi localizing p230–mCherry and golgin-97–mCherry was normalized to the cytosolic redistribution. (C) Quantification of p230 redistribution in control and siRNA Rab6 LPS-activated cells as a ratio of Golgi complex and cytosolic area. * = p<0.05, *** = p<0.001 (pairwise comparisons). (TIF) [file pone.0057034.s002.tif]

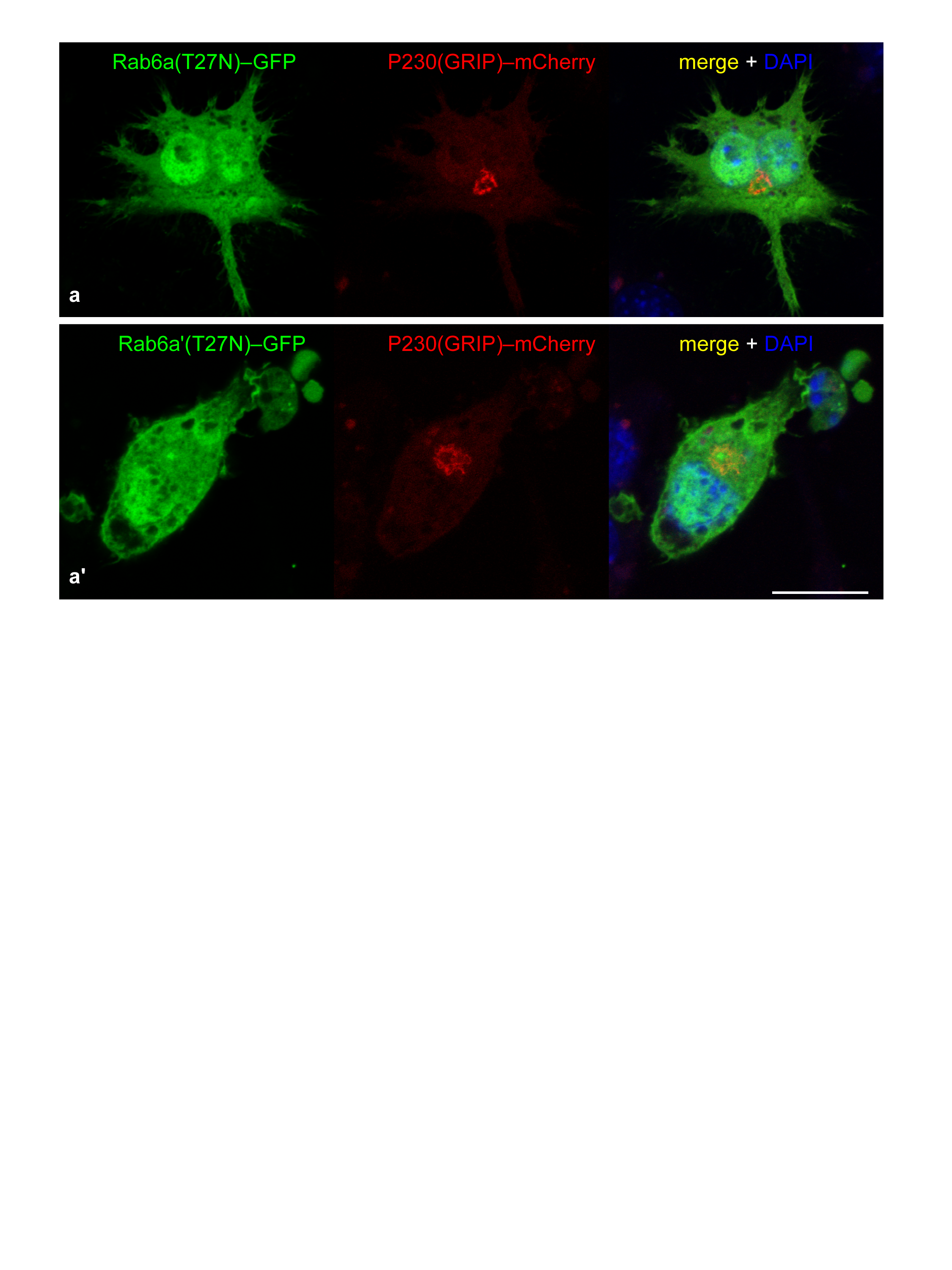

Supplement: Figure S3 — Expression of Rab6a(T27N)–GFP and Rab6a'(T27N)–GFP inhibit Golgi-to-plasma membrane TNF trafficking, but not the Golgi localization of p230. Inactivation of single Rab6a or Rab6a' isoforms by over-expressing the GFP–tagged dominant negative (T27N) proteins resulted in an inefficient cytosolic redistribution of p230, which was for siRNA Rab6. Original optical magnification 63X. Bar: 10 µm. (TIF) [file pone.0057034.s003.tif]
